# A review on antibiotic and non-antibiotic decolonization strategies of multidrug-resistant bacteria in the gastrointestinal tract

**DOI:** 10.1007/s15010-025-02718-2

**Published:** 2026-01-23

**Authors:** Tobias Weirauch, Dafna Yahav, Itay Zahavi, Silvia Würstle, Maria J. G. T. Vehreschild

**Affiliations:** 1 Department II of Internal Medicine, Infectious Diseases, Goethe University Frankfurt, University Hospital Frankfurt, Frankfurt am Main, Germany; 2 Infectious Diseases Unit, Sheba Medical Center, Ramat-Gan, Israel; 3 Faculty of Medical & Health Sciences, Tel-Aviv University, Ramat-Aviv, Tel-Aviv, Israel; 4 School of Public Health, Gray Faculty of Medical and Health Science, Tel Aviv University, Tel Aviv, Israel

**Keywords:** Gut decolonization, Multidrug-resistant organisms, Fecal microbiota transfer, Bacteriophage, Phage, Phage therapy

## Abstract

The escalating global threat of antimicrobial resistance represents a critical challenge for contemporary medicine. Intestinal colonization by multidrug-resistant organisms (MDROs) is increasingly identified as a primary driver of hospital-acquired infections across various patient cohorts. While localized eradication via non-absorbable antibiotics was once viewed as a viable strategy, clinical evidence has failed to demonstrate its efficacy. Consequently, attention has shifted toward microbiome-modulating interventions, such as fecal microbiota transfer (FMT), probiotics, and live biotherapeutic products (LBPs), which have shown potential in preliminary studies. However, current evidence remains fragmented and lacks the support of large-scale randomized controlled trials (RCTs). This review critically assesses both traditional and novel decolonization methods and features a comprehensive summary of clinical studies to highlight existing research gaps. A notable limitation of this analysis is the absence of a formal methodological quality assessment for the included studies. Ultimately, definitive conclusions remain elusive, necessitating future large-scale, pathogen-specific RCTs to validate these emerging approaches.

**Graphical abstract:**

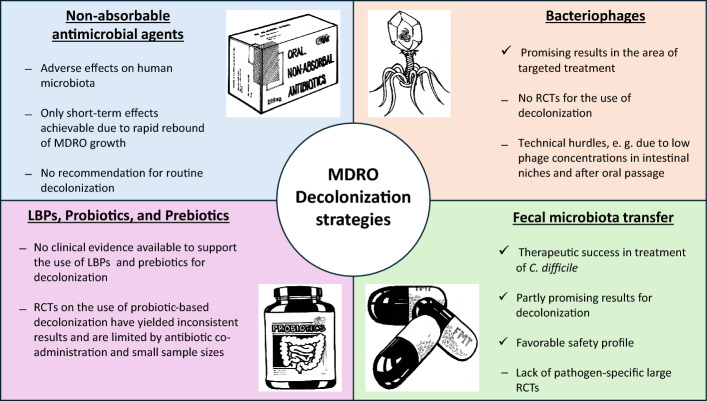

## Introduction

For the year 2050, O'Neill et al. [1] outlined a scenario involving 50 million annual deaths and cumulative damage to the global economy amounting to 100 trillion USD due to antimicrobial resistance (AMR). According to the WHO Global Antimicrobial Resistance and Use Surveillance System report from 2022, AMR is considered one of the top ten global health threats [2]. Bacterial AMR was already estimated to have directly caused 1.27 million deaths worldwide in 2019 [3]. Over the decades, a wide variety of gram-positive and gram-negative bacteria have developed diverse mechanisms of resistance, resulting in a currently uncontrolled problem.

The human gastrointestinal tract (GIT) hosts a complex and dynamic variety of commensal and pathogenic microorganisms, both of which naturally harbor antibiotic resistance genes [4]. Initial colonization of the GIT occurs within hours after birth, typically by facultative anaerobes such as *Escherichia coli*, *Enterococcus*, and *Staphylococcus* species. These early colonizers reduce oxygen levels, enabling the subsequent dominance of obligate anaerobes including *Bifidobacterium*, *Bacteroides*, and *Clostridium* species [5]. The developing gut microbiota provides colonization resistance by competing for nutrients and niches, producing antimicrobial metabolites, and reinforcing the intestinal barrier and immune system. However, within a single host, horizontal gene transfer through bacterial transformation, transduction, or conjugation can lead to the acquisition of antibiotic resistance genes by a broad range of bacterial species in the host’s intestinal microbiota [6–8]. In comparison with other ecosystems, the rate of gene transfer is particularly high in the GIT.

From a clinical perspective, this reservoir is critical because human carriers of MDRO may subsequently develop invasive and potentially lethal infections [9–14]. *Vancomycin-resistant enterococci* (VRE) and carbapenem-resistant *Enterobacteriaceae* (CRE) have spread rapidly and become endemic in many global regions [15–17]. Due to prolonged asymptomatic carriage in the GIT, these pathogens are increasingly affecting vulnerable patient groups such as immunosuppressed individuals and those who have received organ transplants. The increased risk in these groups arises from frequent contact with medical facilities, more frequent invasive interventions, and higher antibiotic exposure [18–20]. Depending on bacterial and host factors, the median duration of ESBL/CRE carriage has been reported to be at least 80 days, with ~ 35% of patients remaining colonized at 1 year [21]. Hence, effective strategies are sought that can counteract asymptomatic colonization to avoid subsequent complications.

Given the increasing realization that antibiotic stewardship and infection control measures alone cannot fully prevent the spread of AMR, new therapeutic approaches are gaining traction. Since the role of the human gut microbiota for human health has been highlighted, it has become a central focus of scientific interest. The physiological composition of the gut microbiota has been shown to play a key role in maintaining colonization resistance and potentially reducing the spread of MDROs [22]. As a result, several therapeutic strategies aimed at restoring a healthy gut microbiome have emerged. Individual therapeutic successes have already been achieved with fecal microbiota transfer (FMT), probiotics, and bacteriophage applications. However, the current data are insufficient to draw definitive conclusions. This review aims to provide an overview on the evidence on the use of different decolonization strategies of MDRO from the gut.

## Methods

The primary objective of this review was to map the existing literature on MDRO gut decolonization with antibiotic and non-antibiotic alternatives. Eligible studies included original research articles, case reports, and clinical trials. Inclusion criteria encompassed all study designs involving all human subjects, provided they reported outcomes related to safety and efficacy. As this is a narrative review, no formal systematic review protocol was registered. Articles not available in english and reviews or editorials without primary data were excluded. A comprehensive literature search was conducted in PubMed, web of science, and cochrane library between January and August 2025. The following search terms were used in combination with Boolean operators: MDRO, gut decolonization, VRE decolonization, CRE decolonization, fecal microbiota transfer, live biotherapeutic products, probiotics, bacteriophages. Titles and abstracts were screened independently by two reviewers (one from Germany and one from Israel). A standardized data extraction form was developed and piloted. Data were independently extracted by two reviewers and included information on the authors and year of publication, the number of recipients and controls, the study design, the target organism(s), the type of treatment, the route of administration, and the outcomes assessed, such as decolonization efficacy and adverse events or safety. The results were synthesized descriptively. A narrative summary and accompanying tables were used to present the data. As this study involved analysis of published literature only, ethical approval was not required. As this is a narrative review, no formal systematic review protocol was registered.

## Decolonization approaches

### Non-absorbable antimicrobial agents

Due to the lack of effective decolonization options using systemic antibiotics, combinations of oral non-absorbable antimicrobial agents have been considered as potential decolonization approaches for decades [23]. Since their effects are limited to the GIT, adverse systemic side effects or toxicity we thought to be minimized. However, their localized action not only inhibits bacterial growth, but also modulates the gut microbiome.

We identified two double-blind, randomized, placebo-controlled trials on VRE decolonization [24, 25]. Wong et al. investigated oral ramoplanin at doses of 100 mg (n = 21) and 400 mg (n = 20), which resulted in 81% (17/21) and 90% (18/20) eradication after 7 days, respectively, but only 21% (6/21) and 41% (7/17) eradication after 14 days, suggesting an early rebound of VRE [24]. Mondy et al. tested oral zinc bacitracin in a very small intervention group (n = 6), which achieved 33.3% (2/6) eradication after three weeks [25]. The results of Weinstein et al. are notable, as a combination of bacitracin and doxycycline was inferior to no treatment during the 130 day follow-up (62.5% vs. 60%), although the study was also small, with only 15 patients in the intervention group [26].

In contrast, two double-blind, randomized, placebo-controlled trials have investigated interventions for ESBL-E/KP colonization [27, 28]. Dimitriou et al. found that oral administration of colistin, gentamicin, and fosfomycin offered no benefit over placebo after a 42 day follow-up (38.9%; 7/18 vs. 37.3%; 3/11) [27]. A larger trial, with 29 patients in the intervention group, reported slightly higher eradication rates for a combination of oral colistin, neomycin, and, when applicable, nitrofurantoin after 28 days (51.9%; 14/27 vs. 37%; 10/27) [28]. Notably, both studies observed a decline in eradication rates from the first to the second follow-up, highlighting potential challenges in sustaining decolonization over time. The largest trial reporting eradication rates was published by Farinas et al. [29]. They investigated oral colistin combined with neomycin for MDR *Enterobacteriaceae* over 30 days, observing eradication rates of 45.3% (24/53) in the intervention group versus 26.3% (14/52) in the placebo group [29]. An overview of all identified studies on the use of non-absorbable antimicrobial agents to reduce MDRO colonization is presented in Table 1. These studies are typically small and often lack control groups, resulting in low statistical power. However, these few studies suggest that the beneficial effects of non-absorbable antibiotics are likely to be short-lived, with MDRO loads recovering afterward. Most studies have shown a rapid rebound of VRE after completing the antibiotic regimen [24, 26, 30]. For non-absorbable antibiotic use in gram-negative bacteria (CRE, ESBL-E) decolonization, similar effects were observed, even when accompanied by additional measures [27, 28, 31].

**Table 1:**
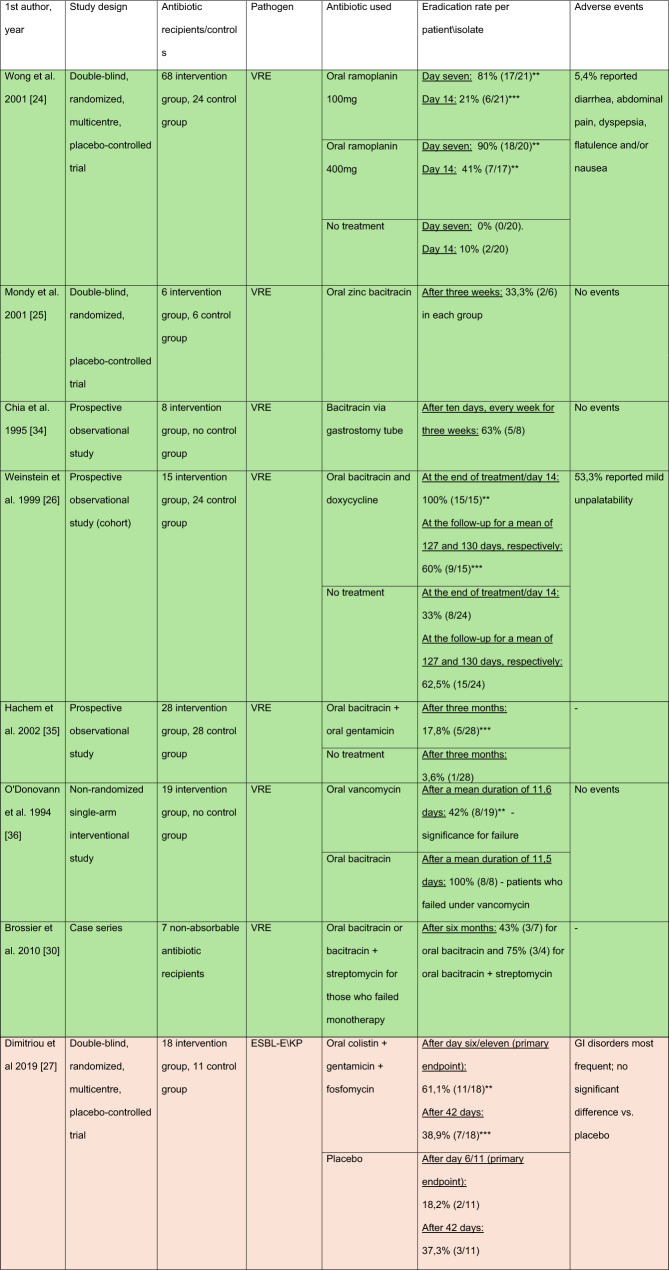

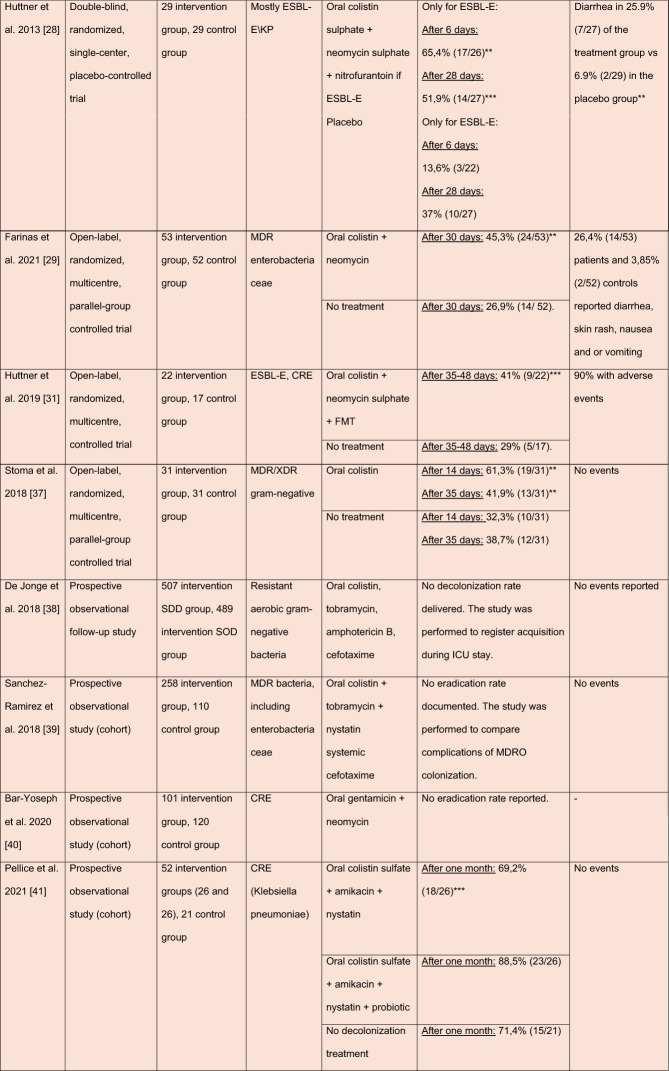
Summary of non-absorbable antibiotic use in MDRO gut colonization, partly adopted from [32, 33]

*CRE* carbapenem-resistant enterobacteriaceae, *ESBL*-*E* extended-spectrum beta-lactamase-producing enterobacteriaceae, *ESBL*-*KP* extended-spectrum beta-lactamase-producing klebsiella pneumoniae, *FMT* fecal microbiota transplant, *GI* gastrointestinal, *ICU* intensive care unit, *MDR* multidrug-resistant, *MDRO* multidrug-resistant organisms, *SDD* selective digestive decontamination, *SOD* selective oropharyngeal decontamination, *VRE* vancomycin-resistant enterococcus, *XDR* extensively drug-resistant

Green: VRE; Light green: other gram-positive bacteria; Light orange: gram-negative bacteria (MDR Enterobacteriaceae, ESBL-E, CRE, MDR/XDR gram-negative); Light blue: Mixed gram-positive and gram-negative bacteria

*The table does not claim to provide a comprehensive overview of the studies ‘ complexity

**Significant difference\change compared to control and\or baseline

***Non-significant difference\change compared to control and\or baseline

### Fecal microbiota transfer

In fecal microbiota transfer (FMT), the microbiome from healthy, carefully screened donors is transferred into the patient’s intestines via methods such as rectal enema, nasogastric/duodenal tubes, colonoscopic application or encapsulated preparations. FMT may restore the diversity and function of the commensal microbiota, thus promoting regulation and resistance to pathogenic bacterial species [42, 43]. Based on success in the non-pharmacological secondary prevention of recurrent *Clostridioides difficile* infections (CDI), where studies indicate significantly higher response rates with traditional FMT (71%) compared to fidaxomicin (33%, p = 0.009) or vancomycin (19%, p = 0.001) alone [44], FMT has also been considered as a strategy for decolonizing patients with MDROs. Although the clinical utility of FMT for different MDROs has not yet been appropriately quantified, FMT has been shown to downregulate the expression of resistance genes, especially VanA, which may lead to MDRO decolonization [45].

A 2019 literature review of FMT use for MDROs showed a cumulative decolonization rate of 68.2% (30/44) for gram-positive bacteria (*methicillin-resistant Staphylococcus aureus* (MRSA); *vancomycin-resistant Enterococci* (VRE)) and 70.6% (72/102) for gram-negative bacteria (carbapenemase-producing *Klebsiella pneumoniae*; carbapenem-resistant *Acinetobacter baumannii*; carbapenem-resistant *Enterobacteriaceae* (CRE); carbapenem-resistant *Pseudomonas aeruginosa*; extended-spectrum beta-lactamase-producing *Enterobacteriaceae*; *Stenotrophomonas maltophilia*) [46]. However, this review included many uncontrolled case series and prospective studies with small sample sizes. Furthermore, the efficacy of FMT for eradicating MDROs varied between bacterial classes. A 2022 systematic review focusing on carbapenem-resistant *Enterobacteriaceae* (particularly *Klebsiella pneumoniae* and *Escherichia coli*) included 112 FMT recipients, of whom 78.7% were decolonized after 6–12 months [47]. A review by Saha et al. analyzed 21 studies involving 192 patients with MDRO colonization or infection treated with FMT [48]. The authors found that, among studies with low to moderate risk of bias, eradication rates ranged from ~ 37.5 to 87.5%, suggesting FMT may effectively decolonize MDROs and possibly prevent recurrent infections, with a generally favorable safety profile [48]. The studies included encompassed a broad range of pathogens, including MRSA, VRE, CRE, extended-spectrum β-lactamase–producing *Enterobacteriaceae*, and additional antibiotic-resistant gram-positive and gram-negative organisms [48].

The only published randomized controlled trial on this topic focused exclusively on multidrug-resistant *Enterobacteriaceae* (ESBL-E/CPE), but it included only a small number of cases, and no significant difference was found between the FMT group (41%; 9/22) and the control group (29%; 5/17) after 35–48 days follow up [49]. FMT was delivered by nasogastric tube or oral capsule [49]. Another small RCT evaluated FMT (enema) for decolonization of MDROs (VRE, CRE, EBSL) in kidney transplant recipients [50]. The authors reported an eradication rate of 67.7% (4/6) in the intervention group, whereas no eradication was observed in the control group (0%; 0/5) [50].

Only a small case series (n = 5) was identified for FMT in MRSA decolonization, reporting 100% eradication after 12 weeks [51]. For other gram-positive bacteria such as VRE, a prospective open-label interventional study (n = 11, no control group) administered FMT via enema, and the six-month follow-up demonstrated an eradication rate of 72.7% (8/11) [52]. We identified two additional small studies on VRE (n = 3–8), both without control groups, reporting eradication rates ranging from 0% to 87.5% [53, 54].

Furthermore, several studies on gram-negative MDROs with lower methodological quality -mostly prospective or retrospective observational designs- showed heterogeneous outcomes, but only two included a control group. Some studies examined both VRE and CRE; however, only the prospective, non-randomized interventional study by Shin et al. included a control group [55].

The results of our literature search, including all smaller studies with information on the number of recipients, FMT administration route, reported eradication rates, and adverse events, are presented in Table 2. Studies are categorized by gram-positive and gram-negative bacteria and organized according to their level of evidence.

**Table 2:**
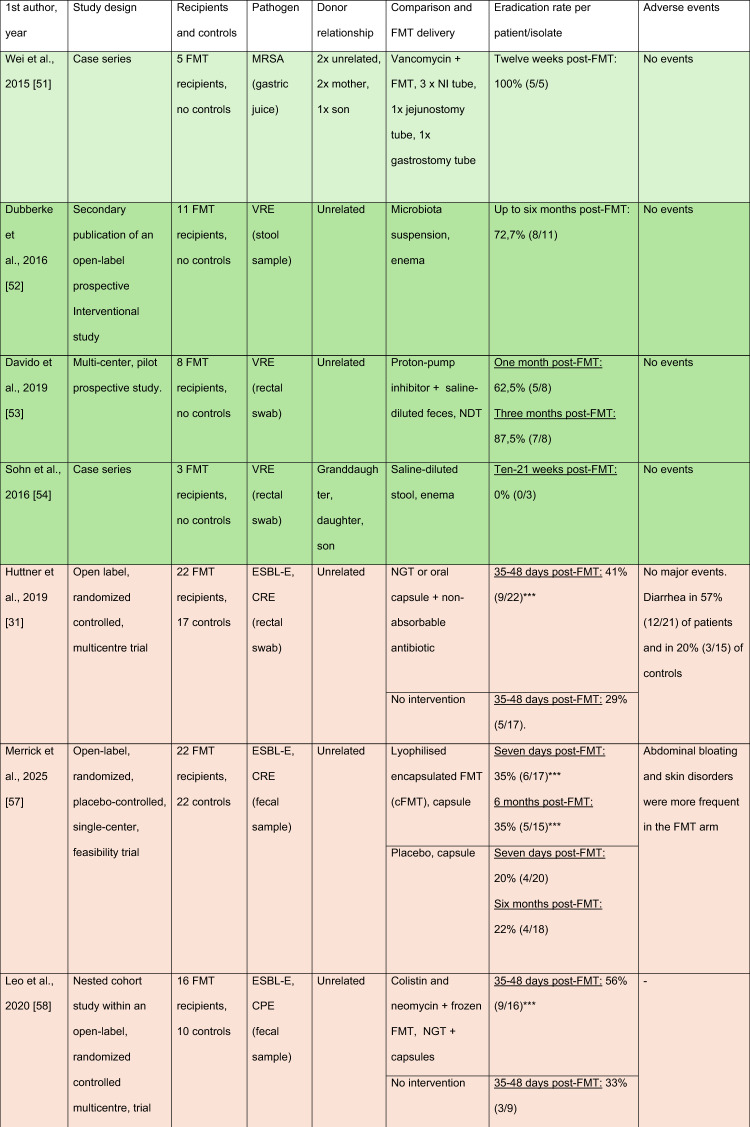

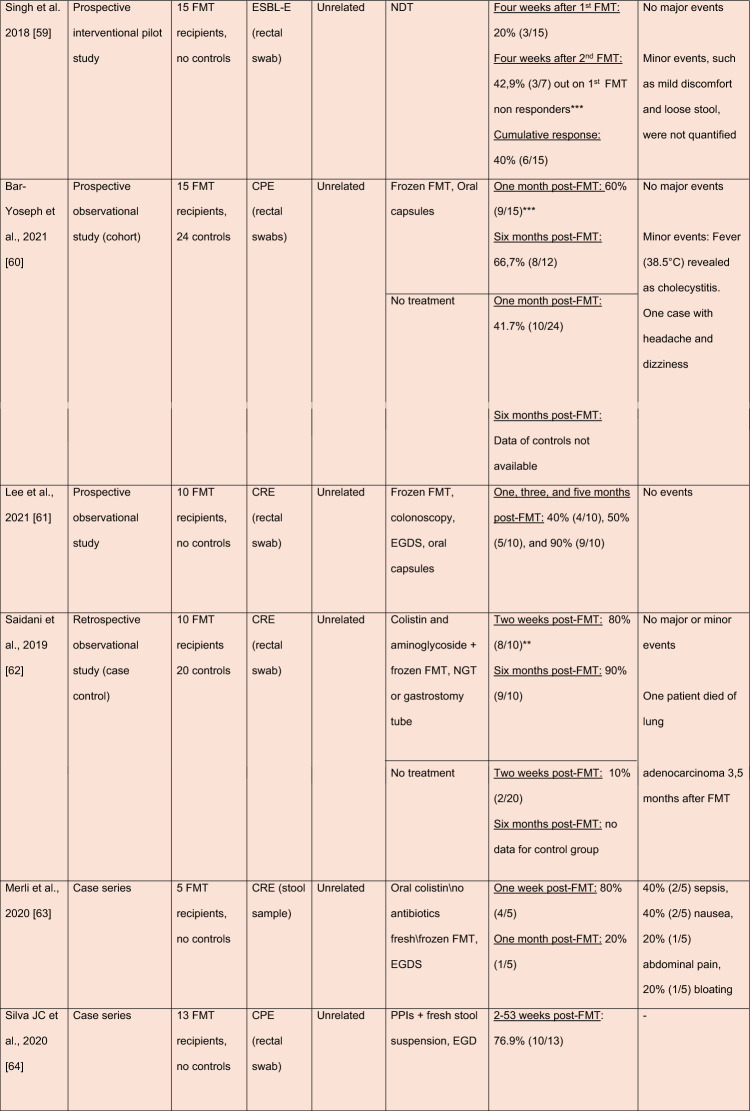

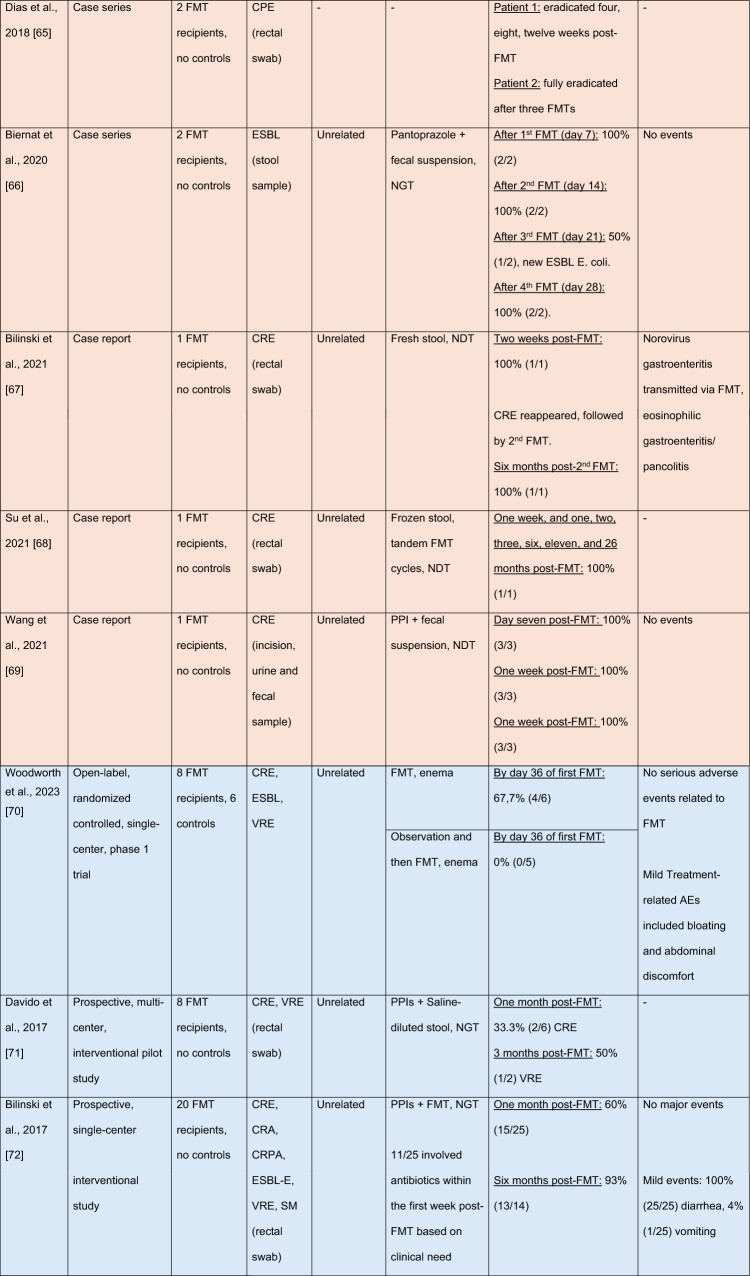

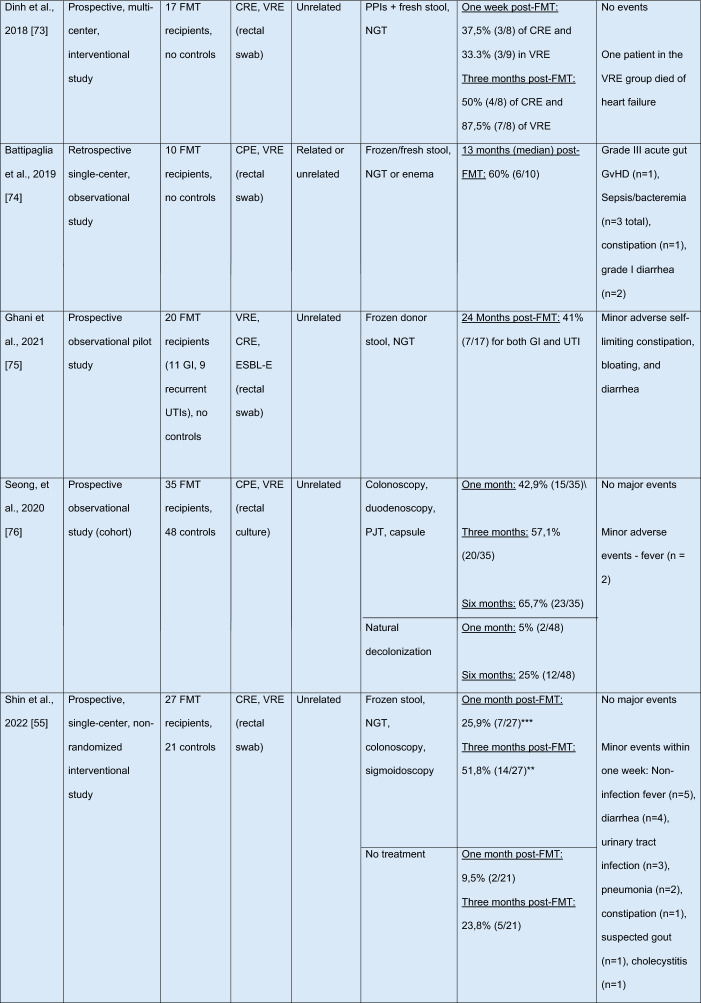
Summary of FMT in MDRO, partly already listed [47, 48, 56]*

*CPE* carbapenemase-producing enterobacteriaceae, *CRA* carbapenem-resistant acinetobacter, *CRE* carbapenem-resistant enterobacteriaceae, *CRPA* carbapenem-resistant pseudomonas aeruginosa, *EGD* esophagogastroduodenoscopy, *EGDS* esophagogastroduodenoscopy; *ESBL*-*E* extended-spectrum beta-lactamase-producing enterobacteriaceae, *FMT* fecal microbiota transplantation, *MDR* multi-drug resistant, *MDRO* multi-drug resistant organisms, *MRSA* methicillin-resistant staphylococcus aureus, *NDT* nasogastric/nasoduodenal tube, *NGT* nasogastric tube, *Ni* nasointestinal, *PJT* percutaneous jejunostomy tube, *SM* stenotrophomonas maltophilia, *UTI* urinary tract infection, *VRE* vancomycin-resistant enterococci, *XDR* extensively drug-resistant

Green: VRE; Light green: other Gram-positive bacteria; Light orange: Gram-negative bacteria (MDR Enterobacteriaceae, ESBL-E, CRE, MDR/XDR gram-negative); Light blue: Mixed Gram-positive and Gram-negative bacteria

*The table does not claim to provide a comprehensive overview of the studies’ complexity

**Significant difference\change compared to control and\or baseline

***Non-significant difference\change compared to control and\or baseline

### Live biotherapeutic products (LBP), probiotics, and prebiotics

LBPs are defined by the U.S. food and drug administration (FDA) and the European medicines agency (EMA) as a biological product that (1) contains live organisms, such as bacteria; (2) is applicable for the prevention, treatment, or cure of a disease or condition in humans; and (3) is not a vaccine. The use of LBPs for the prevention of recurrent *Clostridioides difficile* infections has shown positive effects in a randomized, placebo-controlled phase II trial [77]. Despite the high clinical need, however, there are currently no reliable studies on the use of LBPs in the context of intestinal decolonization of MDROs. As a result, no definitive conclusions can be drawn on this issue at present.

Probiotics, on the other hand, are defined by the International scientific association for probiotics and prebiotics (ISAPP) as ‘live microorganisms which, when administered in adequate amounts, confer a health benefit on the host. They contain one or more strains of microorganisms such as *Lactobacillus*, *E. coli*, *Enterococci*, and yeast strains. Their potential therapeutic benefits arise from the production of bioactive molecules (enzymes, bacteriocins, hydrogen peroxide), modulation of the mucosal epithelial and immune response, and the strengthening of the epithelial barrier [78, 79]. Table 3 provides an overview of the main differentiating criteria between live biotherapeutic products (LBPs) and probiotics.

**Table 3:**
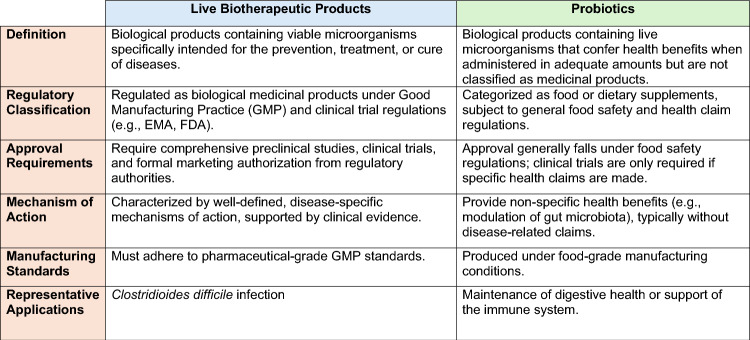
An overview of the main differentiating criteria between live biotherapeutic products and probiotics

#### Probiotics for decolonization of gram-negative bacteria

The evidence on decolonization of gram-negative bacteria is heterogenous. Boudeau et al. could not found in vitro benefits of *E*. *coli* Nissle 1917 [80]. *Escherichia coli* Nissle 1917 was also evaluated in a placebo-controlled study but showed no effect on the decolonization rate of patients with norfloxacin-resistant *E*. *coli* strains in stool [81]. Additionally, two studies evaluating probiotics for the decolonization of ESBL-E/CRE showed mixed results. One small randomized controlled study assessing the daily intake of eight live bacterial strains (Vivomixx®) for decolonization of ESBL-E carriers found no significant effect (p = 0.24), although more participants in the probiotic group met the elimination criteria than in the control group [82]. At 1 year follow up, 12.5% (5/40) of probiotic treated patients achieved decolonization, compared to 5% (2/40) in the control group [82]. Another small study by Choi et al. evaluated a protocol including *Bacillus subtilis* and *Enterococcus faecalis* for decolonizing CRE carriers, with success in 9/13 (69.2%) patients [83], but this study lacked a control group.

#### Probiotics for decolonization of gram-positive bacteria

An open-label follow-up study of newborns found that 21 out of 22 patients were free of VRE within 6 months of treatment with *Lactobacillus rhamnosus* GG [84]. A larger study (n = 65) by Szachta et al. [85] in a pediatric group found a significant decrease in VRE colonization three weeks after probiotic application. However, smaller studies and case series show inconsistent results [86, 87], as summarized by Jain et al. [88]. Three double-blind, randomized, placebo-controlled trials on the use of probiotics in the decolonization of VRE were identified. Doron et al. [89] found no effect from *Lactobacillus rhamnosus* GG on VRE colonization after 56 days. Similar observations were made by Rubin et al. who compared a VRE decolonization strategy with *Lactobacillus rhamnosus* GG to placebo [90]. However, a randomized controlled trial by Manley et al. [91] reported that 11/11 patients cleared VRE 3 weeks after *Lactobacillus rhamnosus* GG treatment. Furthermore, we identified three randomized, double-blind, placebo-controlled trials evaluating the potential of probiotics in decolonization of MRSA. Warrack et al. demonstrated a 50% reduction in MRSA intestinal colonization with *Lactobacillus rhamnosus* HN001 [92]. Similarly, *Lactobacillus rhamnosus* HN001 led to an 83% reduction in MRSA odds in stool samples [93]. However, in both studies the difference of MRSA reduction was not significant compared with placebo [92, 93]. Additionally, *Bacillus subtilis* MB40 reduced MRSA by 96.8% while CFU were analyzed in stool [94]. However, it should be noted that intestinal colonization by MRSA is relatively rare and its clinical relevance in this setting questionable, with only a few reported cases of clinically significant conditions such as MRSA colitis [95].

Rahman et al. [96] summarized clinical outcomes of various probiotics used for gut decolonization of different species. In total, they reported a significant benefit of probiotics for the decolonization of MRE, whereas no meaningful effect was observed for VRE. Probiotics were significantly less effective in decolonizing VRE compared with other pathogens [96]. Overall, there is a lack of data from well-designed randomized controlled trials on the use of probiotics for the decolonization of MDROs. The few available studies are often confounded by co-administration of antibiotics. Only 3 studies were entirely free of antibiotic exposure during the probiotic intervention [83, 86, 94].

In contrast, all remaining studies allowed, mentioned, or could not exclude concomitant antibiotic intake during the probiotic intervention. This likely contributes to the substantial heterogeneity observed across the reported results.

Furthermore, there was no evidence that probiotics can prevent VRE or antibiotic-resistant gram-negative bacteria acquisition [97, 98]. The results of our literature search on probiotics are presented in Table 4.

**Table 4:**
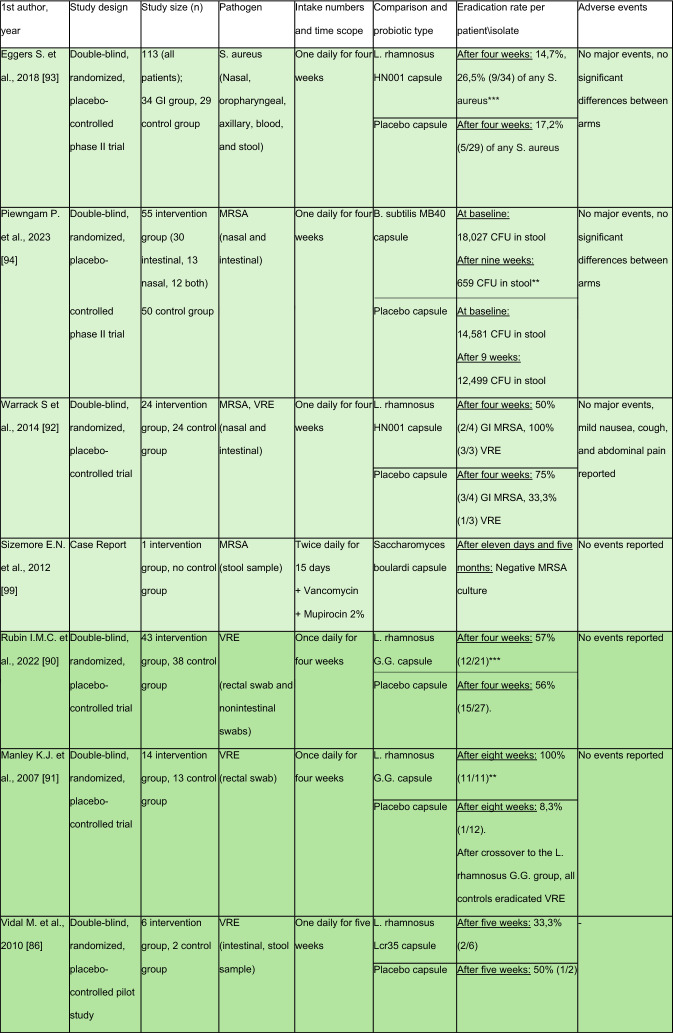

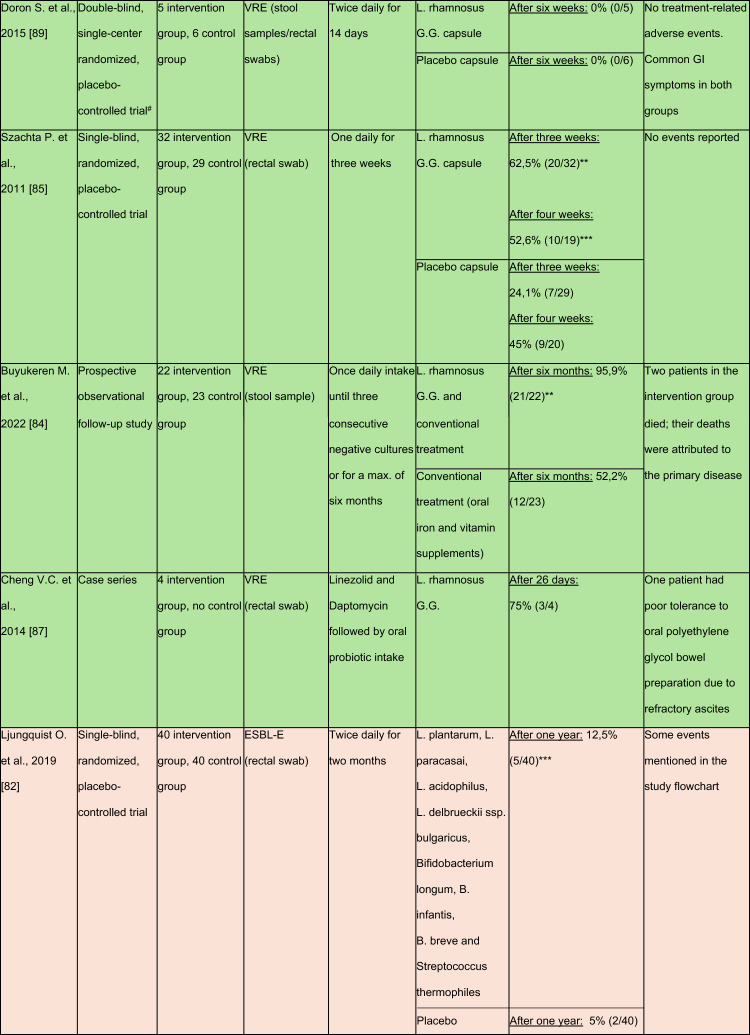

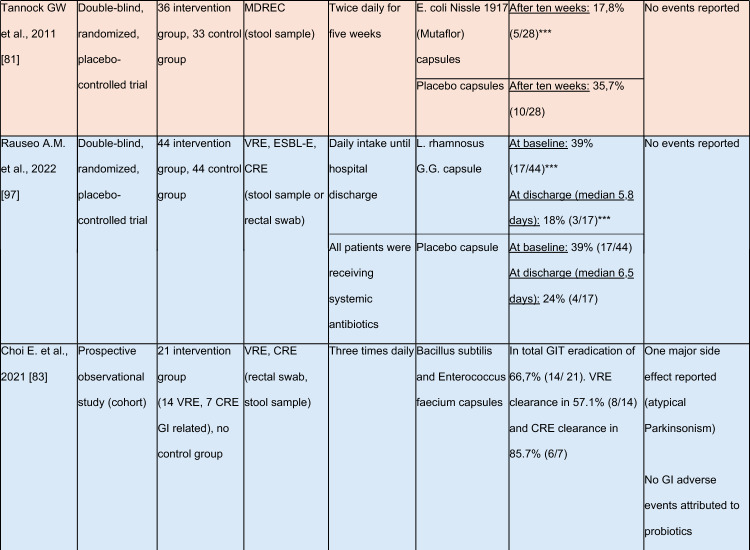
Summary of probiotic use in MDRO, partly already listed [96]*

*ARE* ampicillin-resistant enterococcus faecium, *CRE* ampicillin-resistant enterococci, *C*. *diff* clostridioides difficile, *CFU* colony-forming unit, *CRE* carbapenem-resistant enterobacteriaceae, *E*. *coli* escherichia coli, *ESBL* extended-spectrum beta-lactamase, *ESBL*-*E* extended-spectrum beta-lactamase-producing enterobacteriaceae, *GI* gastrointestinal, *L* Lactobacillus, *MDREC* multidrug-resistant escherichia coli, *MDRO* multi-drug resistant organisms, *MRSA* methicillin-resistant staphylococcus aureus, *VRE* vancomycin-resistant enterococci, *XDR* extensively drug-resistant

Green: VRE; Light green: other gram-positive bacteria; Light orange: gram-negative bacteria (MDR Enterobacteriaceae, ESBL-E, CRE, MDR/XDR gram-negative); Light blue: mixed gram-positive and gram-negative bacteria

*The table does not claim to provide a comprehensive overview of the studies’ complexity

**Significant difference/change compared to control and/or baseline

***Non-significant difference/change compared to control and/or baseline

^#^Planned sample was n = 38 (19 per group); study terminated early due to slow accrual and lack of efficacy

Prebiotics, such as fructans and galactans (e.g., inulin), are non-digestible food components that selectively stimulate the growth or activity of beneficial gut bacteria. Previous studies have shown that these carbohydrates can promote the growth of *Lactobacillus* and *Bifidobacterium* strains [100]. In vitro work with the extracellular polysaccharide of *Weissella cibaria* has shown that prebiotics can regulate growth, antibacterial properties, and antibiofilm activity of *Lactobacillus rhamnosus* [101]. Indirectly, these effects could potentially be used for intestinal decolonization of MDROs. However, no studies have specifically examined the effects of prebiotics on MDROs in humans. Since only indirect benefits are currently proven, further research is urgently needed to evaluate decolonization potential.

Salomao et al. [102] evaluated a symbiotic product (including a combination of probiotics: *Lactobacillus bulgaricus, Lactobacillus rhamnosus,* and prebiotics: fructo-oligosaccharides) for decolonization of various gram negative MDROs and found no beneficial effect of the product compared to placebo in either decolonization or prevention of subsequent clinical infection Table 5.

**Table 5:**
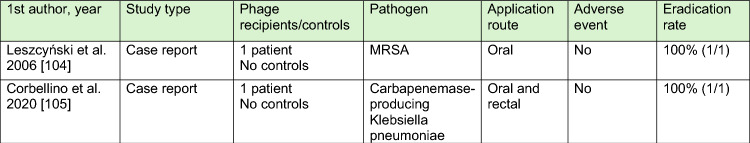
Summary of phage applications in MDRO gut decolonization. Key aspects from each study shown. The table does not claim to give a complete overview of the complexity of the studies

*MRSA* methicillin-resistant staphylococcus aureus

### Bacteriophages

As the incidence of carriage and infection with MDROs continue to rise, bacteriophages (phages) have emerged as a promising therapy option for decolonization. Phages are viruses that specifically target bacteria, executing their antibacterial effect via the lytic cycle, during they infect bacterial cells, replicate, and lyse the host. Their capacity to self-replicate at the site of infection enables sustained reduction of pathogenic bacterial populations. This specificity diminishes disruption to the commensal microbiota—a significant advantage over broad-spectrum antibiotics. While multiple in vivo decolonization attempts have demonstrated that phage administration is well tolerated, clinical application in human for gut decolonization remains scarce [103]. One case report documented the successful eradication of methicillin-resistant *Staphylococcus aureus* (MRSA) intestinal carrier status in a 30 year-old healthcare worker using a protocol of a four-week phage therapy [104]. An oral preparation comprising three highly effective anti-MRSA phage strains was used. Decolonization success was observed microbiologically by rectal swabs [104]. Another case report documented the successful eradication of a multidrug-resistant, carbapenemase-producing *Klebsiella pneumoniae* isolate following oral and intra-rectal therapy with a custom-made, lytic bacteriophage preparation [105].

At present, no clinical trials have been identified that directly investigate the efficacy of gut decolonization of MDROs. Nonetheless, ongoing research may yield insights or indirect implications relevant to the decolonization of MDROs. Prominent examples are the oral administration of phages in patients scheduled for allogeneic hematopoietic stem-cell transplantation and harboring fluoroquinolone-resistant *E. coli* (NCT06938867), the oral administration of phages against adherent invasive *E. coli* to patients with inactive Crohn’s disease (NCT03808103), and the oral administration of phages to reduce or eliminate vancomycin-resistant *Enterococcus* spp. (NCT05715619). Other studies focus mainly on aspects other than gut decolonization or were performed in animal models and thus were not considered in the present analysis.

## Discussion

This review provides a comprehensive and structured overview of all major strategies currently investigated for MDRO decolonization of the GIT, including non-absorbable antimicrobials, FMT, probiotics and bacteriophages. By tabular summary of all relevant studies, study design, sample size, addressed pathogens, characteristics of intervention, clinical outcomes, and safety data across these interventions, the review highlights both the potential and the limitations of each approach. The comparative perspective allows clinicians and researchers to better understand which strategies are supported by stronger evidence and where knowledge gaps remain. Overall, the review aims to support clinical decision-making and guide future research priorities in the rapidly evolving field of MDRO decolonization.

### Summary of evidence

Our scoping review evaluates both antibiotic and non-antibiotic approaches for gut decolonization of MDROs. For each analyzed strategy, we identified key findings:

1. Non-absorbable antimicrobial agents often lead to a rapid rebound of MDROs following treatment, resulting in a lack of sustained efficacy [106]. Consequently, their use is not recommended for routine decolonization [107].
2. FMT has shown promising results [46, 47]. However, large randomized controlled trials (RCTs) that distinguish between specific MDRO pathogens and assess the specific effects of antibiotic induction and dosage on microbial engraftment are still needed.
3. Probiotics and prebiotics lack robust scientific evidence for MDRO decolonization. The few available studies are frequently confounded by the co-administration of antibiotics, making it difficult to draw solid conclusions about their independent efficacy.
4. Bacteriophages may represent an alternative for modulating the intestinal microbiota by selectively eliminating pathogenic bacteria. However, currently only two case reports are available in the specific context of MDRO decolonization.

Oral non-absorbable antimicrobial agents used for MDRO decolonization can disrupt the gut microbiota, leading to dysbiosis and reduced colonization resistance [27]. This disruption may promote the selection and overgrowth of resistant organisms, potentially worsening the resistance problem. Additionally, repeated or prolonged use can increase the risk of *Clostridioides-difficile*-infection [108]. Overall, the 2019 ESCMID-EUCIC clinical guidelines on the decolonization of multidrug-resistant gram-negative bacteria advise against a general recommendation for decolonization [107]. Our findings support that future research should move away from antibiotic-based approaches, as substantial evidence already highlights their limited and potentially harmful effects. As demonstrated by Weinstein et al. for VRE and Dimitriou et al. for ESBL-E/KP, spontaneous eradication rates may be comparable to or even exceed- those achieved with antimicrobial treatment, particularly depending on the duration of follow-up [26, 27]. Although the VRE eradication rate for oral bacitracin and doxycycline was 100% (15/15) at 14 days, the 130 day follow-up demonstrated similar eradication compared with the no-treatment group (60%, 9/15 vs. 62.5%, 15/24) [26].

When key confounding factors -such as concomitant antibiotic use, small sample sizes, heterogeneous patient populations, and variations in probiotic strains, dosage, and administration -are considered, the effectiveness of probiotics and prebiotics appears minimal, raising doubts about their utility for MDRO decolonization. Robust data could not be obtained for the decolonization of either gram-positive or gram-negative bacteria. The evidence base remains inconsistent in both domains. However, probiotic-mediated intestinal decolonization appears to be more effective against gram-positive bacteria. Some probiotics (e.g. lactic acid bacteria) produce bacteriocins or acids that disrupt bacterial cell membranes or inhibit cell wall synthesis. Both mechanisms access the peptidoglycan layer of gram-positive bacteria [109]. In contrast, gram-negative bacteria are protected by an additional outer membrane with lipopolysaccharides (LPS), which act as a barrier and limit the penetration of bacteriocins and other antimicrobial compounds. Overall, there is a clear need for more high-quality, well-designed studies to guide clinical practice in this area. At least, the available evidence from randomized controlled trials suggests that probiotics are generally safe [110]. However, assessing their effectiveness or adverse events remains difficult due to various confounding factors in MDRO treatment.

In contrast, FMT and bacteriophages appear to be promising alternatives, although many questions remain regarding its standardization and long-term outcomes. Future studies on FMT should focus on large, randomized, controlled trials to better assess the benefits of FMT with respect to specific pathogens. The FERARO feasibility trial investigated the safety and tolerability of encapsulated FMT in patients colonized with ESBL or carbapenemase-producing *Enterobacterales* [57]*.* Gastrointestinal carriage of multidrug-resistant organisms decreased over time in both the FMT and placebo groups, but carriage rates were consistently lower in the FMT arm, which also showed significant increases in microbiome diversity [57]. Another randomized, controlled trial is currently being conducted [111]. FMT is generally considered a safe therapeutic alternative to antibiotics in specific situations. While many patients experience adverse events such as transient diarrhea, nausea, abdominal pain, low-grade fever, bloating, flatulence, and constipation, severe adverse events are rare. In the past, they have been associated with incomplete screening approaches and risks associated with specific delivery routes [112]. A thorough donor screening is mandatory to minimize FMT risks, which includes the exclusion of: (i) potentially pathogenic microorganisms (bacteria, viruses, parasites, fungi); (ii) severe comorbidities (gastrointestinal, neurological, psychiatric, metabolic, autoimmune, oncological); (iii) previous medical treatments (antibiotics, immunosuppressive drugs, PPIs, inpatient hospital stays, etc.); (iv) travel to countries with increased gastrointestinal infection risks; and (v) social factors (drug abuse, sexual risks, etc.) [112]. The FDA has reported individual cases of severe adverse events linked to extended-spectrum beta-lactamase (ESBL)- and Shiga toxin-producing *Escherichia coli* infections, prompting a national alert and a mandate for additional screening for ESBL to prevent future occurrences [113]. Most short-term risks, such as aspiration pneumonia, are related to the delivery method, with upper gastrointestinal FMT being associated with a higher incidence of adverse events (43.9%) compared to FMT via the lower gastrointestinal tract (20.6%) [114].

FMT has been shown to reduce healthcare costs for recurrent CDI were reduced by 42% with FMT [115]. A recent review confirmed the benefit of FMT with respect to reductions in treatment costs and length of inpatient stay compared to current antibiotic strategies used in clinical practice [116]. Based on these data, successful decolonization of MDRO might likewise reduce associated costs. At this point, however, clinical efficacy of this approach remains to be proven.

Gut decolonization by phages remains promising but challenging because the gut’s spatial heterogeneity presents formidable obstacles. Bacteria often reside in protected niches (mucus layer, crypts, biofilms) difficult for luminal phages to access [117–119]. Furthermore, oral administration encounters barriers: the acidic, enzyme-rich stomach environment [120] and host immune responses like secretory IgA [121, 122]. The rapid evolution of phage resistance (e.g., receptor mutation, CRISPR) poses another challenge [123, 124], potentially compromising decolonization despite fitness costs [125–128].

Formulation strategies aim to overcome the hostile gastrointestinal environment and ensure active phages are delivered to colonization sites. Systems are designed to release phages in response to intestinal pH, while mucoadhesive properties can enhance retention. Liposomes may protect phages against degradation [129, 130]. Alginate-based beads, often combined with calcium carbonate, effectively protect phages; in poultry and rodent models, this resulted in higher stability and efficacy [131, 132]. Other potential solutions (e.g., chitosan-coated alginate microspheres, enteric polymer coatings) are in development [133, 134]. Another future approach is fecal bacteriophage transfer [135]. Generally, phage decolonization appears safe, with no significant adverse reactions [103].

While essential, clinical trials remain highly challenging due to host specificity, regulatory hurdles, and patient microbiota variability. For most targets, large-scale trials are not feasible, making targeted preclinical studies and case reports crucial. Continued research combining innovative formulation strategies with designed clinical studies will be necessary to translate phage therapy into a practical tool.

In summary, the choice of decolonization strategy might depend on both the pathogen and patient characteristics. Non-absorbable antimicrobial agents could be considered for short-term reduction of MDROs, particularly in high-risk or preoperative patients, although the effect is often transient and may promote resistance. Fecal microbiota transplantation (FMT) might be preferred in cases of persistent colonization, dysbiosis, or failure of antibiotic therapy. Probiotics might be considered, primarily targeting gram-positive bacteria, while their efficacy against gram-negative MDROs appears limited. However, no clear evidence-based recommendation can be made, particularly regarding bacteriophage therapy, due to the limited available data.

### Limitations

This work has several strengths and limitations. A systematic literature search was conducted, and the completeness of the data was independently verified by researchers from two different faculties. However, the methodological quality of the included studies was not assessed. Furthermore, no formal risk of bias analysis was performed.

The available evidence on MDRO decolonization is limited by substantial heterogeneity in study designs, small sample sizes, and inconsistent outcome definitions across studies. Many interventions were evaluated in observational or pilot settings, which restricts the generalizability of the findings. Follow-up durations were frequently short, preventing robust conclusions about long-term sustained decolonization and safety. Furthermore, standardized protocols for dosing, donor selection (for FMT), and strain specification (probiotics) are still lacking.

## Conclusion

The findings presented in this review are largely consistent with the current understanding of gut microbiota dynamics and MDRO decolonization mechanisms. Interventions such as FMT demonstrate effects that align well with their proposed ability to restore microbial diversity and enhance colonization resistance. Furthermore, the lack of efficacy of certain non-absorbable antimicrobials was summarized by this review and is consistent with the current understanding that this approach can only reduce bacterial density in the GIT. Results from LBP and probiotic studies, however, are still heterogeneous, and the amount of data included in this review is insufficient to draw definitive conclusions.

The era of uncontrolled prescription of antibiotics must come to an end, as it has been shown to be unsustainable. The second step is to implement WHO control and response strategies, with region-specific measures. Finally, there might be great potential, especially in the different types of microbiota and nutrition-based decolonization approaches discussed in this text, namely FMTs, LBPs, probiotics, prebiotics and phage therapy.

Future research should prioritize well-designed randomized controlled trials, harmonized outcome measures, and systematic long-term follow-up to better assess durability, safety, and patient subgroups that benefit most. Therefore, significant investments in research are essential, as current therapeutic options are insufficient to cope with the problem.

## Data Availability

No datasets were generated or analysed during the current study.
