# Publisher Correction: A review on antibiotic and non-antibiotic decolonization strategies of multidrug-resistant bacteria in the gastrointestinal tract

**DOI:** 10.1007/s15010-026-02777-z

**Published:** 2026-04-09

**Authors:** Tobias Weirauch, Dafna Yahav, Itay Zahavi, Silvia Würstle, Maria J. G. T. Vehreschild

**Affiliations:** 1 Department II of Internal Medicine, Infectious Diseases, Goethe University Frankfurt, University Hospital Frankfurt, Frankfurt am Main, Germany; 2 Infectious Diseases Unit, Sheba Medical Center, Ramat-Gan, Israel; 3 Faculty of Medical & Health Sciences, Tel-Aviv University, Ramat-Aviv, Tel-Aviv, Israel; 4 School of Public Health, Gray Faculty of Medical and Health Science, Tel Aviv University, Tel Aviv, Israel

**Publisher Correction: Infection** **https://doi.org/10.1007/s15010-025-02718-2**

The original publication of the article contained errors introduced during typesetting, including an incorrect display of the abstract figure, table formatting issues, and interchanged reference citations.

The publisher apologizes for these errors and the inconvenience.

The original article has been corrected.

